# Adaptability and Persistence of the Emerging Pathogen *Bordetella petrii*

**DOI:** 10.1371/journal.pone.0065102

**Published:** 2013-06-04

**Authors:** Adrian M. Zelazny, Li Ding, Joanna B. Goldberg, Lilia A. Mijares, Sean Conlan, Patricia S. Conville, Frida Stock, Samuel J. Ballentine, Kenneth N. Olivier, Elizabeth P. Sampaio, Patrick R. Murray, Steven M. Holland

**Affiliations:** 1 Laboratory of Clinical Infectious Diseases, National Institute of Allergy and Infectious Diseases, National Institutes of Health, Bethesda, Maryland, United States of America; 2 Microbiology Service, Clinical Center, National Institutes of Health, Bethesda, Maryland, United States of America; 3 Department of Microbiology, University of Virginia Health System, Charlottesville, Virginia, United States of America; 4 National Human Genome Research Institute, National Institutes of Health, Bethesda, Maryland, United States of America; 5 Leprosy Laboratory, Oswaldo Cruz Institute, FIOCRUZ, Rio de Janeiro, Brazil; Ghent University, Belgium

## Abstract

The first described, environmentally isolated, *Bordetella petrii* was shown to undergo massive genomic rearrangements *in vitro*. More recently, *B. petrii* was isolated from clinical samples associated with jaw, ear bone, cystic fibrosis and chronic pulmonary disease. However, the *in vivo* consequences of *B. petrii* genome plasticity and its pathogenicity remain obscure. *B. petrii* was identified from four sequential respiratory samples and a post-mortem spleen sample of a woman presenting with bronchiectasis and cavitary lung disease associated with nontuberculous mycobacterial infection. Strains were compared genetically, phenotypically and by antibody recognition from the patient and from inoculated mice. The successive *B. petrii* strains exhibited differences in growth, antibiotic susceptibility and recognition by the patient’s antibodies. Antibodies from mice inoculated with these strains recapitulated the specificity and strain dependent response that was seen with the patient’s serum. Finally, we characterize one strain that was poorly recognized by the patient’s antibodies, due to a defect in the lipopolysaccharide O-antigen, and identify a mutation associated with this phenotype. We propose that *B. petrii* is remarkably adaptable *in vivo*, providing a possible connection between immune response and bacterial evasion and supporting infection persistence.

## Introduction

The genus *Bordetella* includes nine species. *B. pertussis* and *B. parapertussis* cause whooping cough in humans [1] while *B. bronchiseptica* infects animals, and rarely humans [1]. Four new species have been recently reported in humans: *B. holmesii* [2], [3], *B. trematum* [4], *B. ansorpii* [5], and *B. petrii* [6].

*Bordetellae* express several virulence factors, which are controlled by the master regulator BvgAS [7]. Lipopolysaccharide (LPS) plays a significant role in infection through the generation of protective immunity, resistance to complement-mediated killing, and cytokine induction. *B. pertussis* LPS lacks O-antigen, but it is commonly expressed in *B. parapertussis* and *B. bronchiseptica* [1], which contain the O-antigen biosynthesis locus, *wbm* [8].

Following its environmental isolation [9], *B. petrii* has been linked to mandibular osteomyelitis [6], mastoiditis [10] and more recently chronic pulmonary disease [11]. *B. petrii* has been isolated from CF patients, although its clinical significance is unclear [12], [13]. Whole genome sequencing of the environmental type strain of *B. petrii* identified huge genomic islands, and several virulence factors including filamentous hemagglutinin and BvgAS [14]. Unfortunately, no strains from clinical sources have been sequenced so far. Given its recent recognition, the pathogenicity and clinical importance of *B. petrii* remains unknown.

We describe the sequential isolation of *B. petrii* from four respiratory samples and a post-mortem spleen sample of a woman with bronchiectasis and cavitary lung disease associated with nontuberculous mycobacteria. The successive strains exhibit phenotypic and genetic differences, and dissimilar antibody recognition by the patient. Using mice, we confirm this specificity and strain dependence in the antibody response. Finally, we characterize one strain that exhibited impaired antibody recognition, show it to be defective in the O-antigen portion of LPS, and identify a mutation likely responsible for this phenotype. Our work highlights the adaptability of *B. petrii*, connects immune response to bacterial evasion, and suggests a more pathogenic role for this organism than previously thought.

## Materials and Methods

### Ethics Statement

Use of patient blood was approved by the Institutional Review Board at the National Institutes of Health. The patient signed informed consent. Animal use was approved by the NIAID Animal Care and Use Committee. The program complies with The Guide for the Care and Use of Laboratory Animals (National Research Council) and with the National Institutes of Health OACU ARAC guidelines.

### Bacterial Strains

Five strains of *B. petrii* (*B. petrii* 1–5) were serially obtained from sputum or bronchoalveolar lavage (BAL) (*B. petrii* 1–4), or spleen at autopsy (*B. petrii* 5) of the same patient between May 2006 and January 2007. *B. petrii* type strain was obtained from the American Type Culture Collection (ATCC BAA-461), while the first described clinical strain (mandibular osteomyelitis case) [6], was obtained from the National Collection of Type Cultures (NCTC 13363), London, UK.

Strains were stored at −80°C using Microbank™ storage system (Pro-Lab Diagnostics Round Rock, TX) and grown on sheep blood agar (SBA) for 24–48 h at 37°C prior to use. Growth curves were performed in LB broth with shaking at 37°C and growth was followed by colony forming units (CFU) determinations with serially diluted samples plated onto SBA plates.

### Identification and Susceptibility Testing

Identification was initially attempted with the API 20 NE system (bioMerieux, Inc Hazelwood, MO), standard stains and biochemical tests. Full sequencing of the 16S rRNA gene (MicroSEQ® Full Gene, Applied Biosystems, Foster City, CA) and partial sequencing of the *Bordetella risA* and *ompA* genes [6] were then performed. Susceptibility testing was done using MicroScan microdilution and E-test methods.

### Molecular Typing

The DiversiLab Non-fermenter typing kit (bioMerieux, Inc.) was used for rep-PCR typing. Pulsed-field gel electrophoresis (PFGE) of macrorestricted DNA was performed as described previously [15] with few modifications. *XbaI* digested DNA was separated on a CHEF Mapper system (Bio-Rad, Richmond, CA). Lambda DNA Ladder 48.5 kb–1000 kb plugs (Lonza Basel, Switzerland) was used as reference.

### Immunoblots against Bacterial Components

Five ml of late exponential cultures (OD_600_∼0.8) of the bacterial strains grown in LB broth were spun for 10 min at 8000×*g* to harvest bacterial cells. Bacterial pellets were resuspended in 200 µl of the Bacterial Protein Extraction Reagent (Pierce, Rockford, IL). These cell lysates were then centrifuged at 16000 rpm for 16 min at 4°C and the supernatant fractions removed. Cell pellets were re-extracted once again with same amount of lysis buffer. Supernatant from the two extractions (soluble proteins) and cell debris pellet (insoluble proteins) fractions were retained. Protein concentrations in both soluble and insoluble fractions were measured by Bradford Protein Assay (Bio-Rad, Hercules, CA). Ten µg of protein or LPS per lane were electrophoresed on 12% SDS–PAGE, and transferred to PVDF membranes (Invitrogen, Carlsbad, CA). Control *Escherichia coli* LPS (3 µg/lane) was obtained from Sigma-Aldrich (St. Louis, MO).

The membrane was blocked in TBS buffer containing 5% nonfat milk and 0.05% Tween-20 before incubation with the patient serum (1∶5000 dilution) obtained ∼2 months after isolation of *B. petrii* 3 or mouse serum (1∶200 dilution). After three washes, the membrane was incubated with horseradish peroxidase conjugated sheep anti-human or sheep anti-mouse IgG (1∶10000) (Amersham Biosciences, Little Chalfont, UK), respectively, and blots were developed with ECL Plus (GeHealthcare, Piscataway, NJ).

### LPS Isolation

LPS was isolated from five ml of late exponential cultures (OD_600_ ∼0.8) in LB broth following centrifugation (10 min 8000×*g*) using LPS extraction kit (Boca Scientific, Boca Raton, FL), and the concentration was determined spectrophotometrically (205 nm). LPS was separated by 12% SDS-PAGE and stained using Pro-Q Emerald 300 Lipopolysaccharide gel stain kit (Molecular Probes/Invitrogen, Carlsbad, CA). Limulus amoebocyte lysate (LAL) gel-clot test (Associates of Cape Cod, E. Falmouth, MA), with 1.0 EU/ml sensitivity was used for endotoxin testing.

### Mice

Wild type C57BL/6 mice were inoculated intraperitoneally (ip) three times every two weeks with either bacterial extracts (40 µg protein), live bacteria (10^4^ CFU) or purified LPS (15 to 100 µg). Mice sera were collected by tail bleeding two weeks after the third (last) inoculation.

### Cell Culture and Cytokines Detection

Human peripheral blood mononuclear cells (PBMCs) from normal volunteers were obtained through centrifugation and seeded in culture (1×10^6^) on 12-mm coverslips in complete RPMI medium as described previously [16]. Purified LPS (200 ng/ml) was added for 20 hours after which supernatants were collected and stored at −20°C until cytokine measurements using multiplex bead-based assays (BioRad Laboratories, Hercules, CA).

### Serum Sensitivity Assay

Bacteria grown on SBA were resuspended in 1% proteose peptone-phosphate-buffered saline (PP-PBS) to a concentration of 1×10^7^ CFU/ml. A 100-µl aliquot was combined with an equal volume of 10% normal human serum (diluted in PP-PBS). A 1% PP-PBS (0% serum) solution or heat-inactivated serum (56°C for 1 hour) served as controls. Samples were incubated for two hours at 37°C with shaking and survival assessed by CFU determinations.

### Statistical Analysis

CFU and cytokine data was plotted using InStat/Prism software version 5.0a (GraphPad Software, San Diego, CA) with values expressed as mean ± SEM. Differences between groups were assessed by one-way ANOVA.

### Sequencing

Genomic DNA from *B. petrii* 1, *B. petrii* 3, *B. petrii* 4, and *B. petrii* 5 was sequenced as both fragment and paired end (3 kb-insert) libraries on a 454 FLX pyrosequencer (Roche Branford, CT). Draft genomes were assembled with gsAssembler (v2.3) and annotated with RAST server [17]. Putative LPS synthesis genes were identified by BLAST, using *B. bronchiseptica* cosmid BbLPS1 (AJ007747) as query. The Mauve aligner v2.3.1 [18] was used for alignments and ortholog identification.

Polymorphisms and indels were verified by Sanger sequencing as follows: Primers BP104-151-9184F (5′-ATAGCCTAGACACGAACGTCAGC) and BP104-151-9184R560 (5′-CTTGTGGCGTTCCTGGAC) were used to amplify the region surrounding the deletion noted in *B. petrii* 3. PCR products from our strains and *B. petrii* NCTC 13363 were sequenced by ACGT Inc. (Wheeling, IL).

Genome data were submitted to GenBank under accession number JF933901.

## Results

### Clinical Presentation

A 54 year-old woman presented in 2000 with idiopathic bronchiectasis and mycobacterial infection. When seen at the NIH (February 2006), while on prednisone and antimycobacterial drugs, she reported a one-month history of dry cough, dyspnea and fatigue. Chest CT showed extensive nodular bronchiectasis and cavitary disease. Bronchoscopy yielded *Pneumocystis jiroveci*, *Mycobacterium intracellulare* (MI) and *Aspergillus fumigatus*. Trimethoprim/sulfamethoxasole (TMP/SMX) and voriconazole were added with improvement. In May 2006 she had increased dyspnea, productive cough and worsening of her CT. Sputum on two occasions (two weeks apart) showed heavy growth of MI and moderate growth of a gram-negative rod eventually identified as *B. petrii* (*B. petrii* 1 and *B. petrii* 2). She received several weeks of intravenous meropenem for *B. petrii* and rifampin and intravenous amikacin for MI led to improvement. In July 2006 her CT scan was improved despite persistent heavy growth of MI on sputum culture; there was no growth of *B. petrii*. Clofazimine was added for MI. In August 2006 sputum remained negative for *B. petrii* but continued to grow MI; CT was unchanged. One week later she had severe dyspnea, productive cough and wheezing associated with heavy growth of *B. petrii* (*B. petrii* 3); MI culture was unchanged. TMP/SMX was added for *B. petrii* with improvement. In November 2006 she had worsened dyspnea, cough, and fatigue; sputum yielded heavy growth of MI and light growth of *B. petrii* (*B. petrii* 4). Thoracoscopic lung biopsy (December 2006) for persistent hypoxia had negative microbial stains and cultures but was complicated by a bronchopleural fistula. She died of progressive respiratory failure in January 2007. Lungs at autopsy showed consolidation particularly in the lower lobes, with cavitary, miliary and caseating lesions. Microscopic exam revealed emphysematous changes and extensive granulomatous inflammation with necrosis. Acid-fast bacilli were seen but no other microorganisms; cultures were negative. Her spleen showed mild congestion with negative microbial stains. However, spleen cultures yielded light growth of *B. petrii* (*B. petrii* 5).

### Identification and Susceptibility Testing of *B. petrii*

During the evaluation of bacterial cultures, a gram-negative rod was first isolated in early May 2006 from several media including sheep blood, chocolate, and McConkey agar. The same organism was subsequently isolated two weeks later (*B. petrii* 2) and also in August (*B. petrii* 3), and November (*B. petrii* 4) of 2006 and in January of 2007 (*B. petrii* 5). *B. petrii* 3, *B. petrii* 4 and *B. petrii* 5 displayed distinctly smaller colonies than *B. petrii* 1 and *B. petrii* 2 on SBA.

Following inconclusive identification by standard methods, 16S rRNA full gene and *Bordetella risA* and *ompA* partial gene sequencing were performed. The first two gene sequences matched 100% with the first reported two clinical strains of *B. petrii* [6], [10] while *ompA* sequence matched 100% recently reported serial strains [11].

Antibiotic susceptibility testing [19] showed the following results with minimal inhibitory concentrations (MICs) in µg/ml. *B. petrii* 1 was susceptible to gentamicin (MIC≤4), tobramycin (MIC≤4), amikacin (MIC≤16), meropenem (MIC≤4), imipenem (MIC≤4), levofloxacin (MIC≤2), piperacillin/tazobactam (MIC≤16), ticarcillin/clavulanate (MIC≤16), TMP/SMX (MIC≤2/38) and resistant to aztreonam (MIC>16), ceftazidime (MIC>16), cefepime (MIC>16) and ciprofloxacin (MIC>2). However, resistance to additional drugs was progressively and cumulatively observed in subsequent strains: piperacillin/tazobactam (MIC>64) and ticarcillin/clavulanate (MIC>64) for *B. petrii* 2, levofloxacin (MIC = 4) for *B. petrii* 3 and >4 for subsequent strains, TMP/SMX (MIC>2/38) for *B. petrii* 4 and meropenem (MIC>8) for *B. petrii* 5.

### Typing and Growth Pattern of *B. petrii*

**Figure 1:**
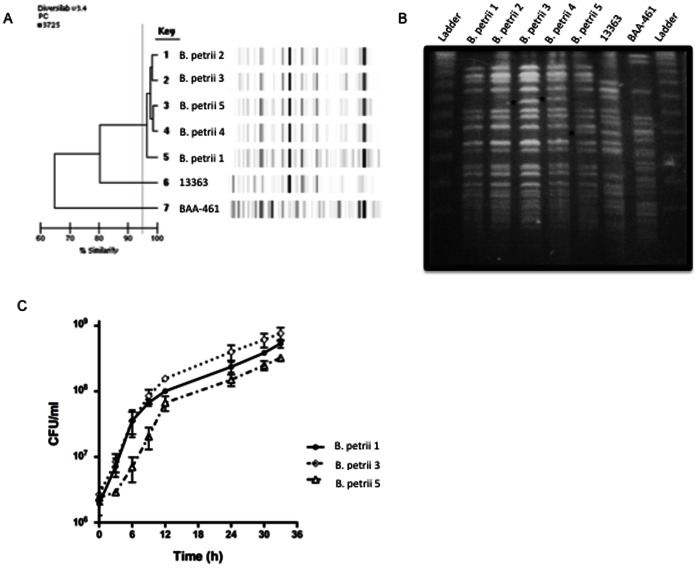
Molecular typing and growth curves of serially isolated strains of *B. petrii.*. (A) The DiversiLab Non fermentor typing kit was used for rep-PCR typing of *B. petrii* using DNA from clinical and reference strains. Amplicons were detected with the Agilent 2100 bioanalyzer (Agilent Technologies, Palo Alto, CA) and data analyzed with the DiversiLab software (version 3.3). Results generated include a dendrogram (left) and virtual gel images (right). (B) Genomic DNA was digested with the restriction endonuclease *XbaI* and separated by PFGE with a CHEF Mapper system. Asterisks indicate band differences among the patient strains. Ladder: Lambda DNA Ladder 48.5 KB–1 MB kb plugs (Lonza). (C) Growth curves were performed on LB broth at 37°C and growth assessed by colony-forming units (CFU) performed with serially diluted aliquots plated on SBA plates. Graph shows mean and SEM from three experiments. *B. petrii* 1–5: strains of *B. petrii* serially isolated from our patient; BAA-461: type strain of *B. petrii* (ATCC BAA-461); 13363: first described clinical strain of *B. petrii* (NCTC 13363).

To assess strain relatedness both rep-PCR and PFGE analysis were used. As shown in Fig. 1A, the five serial strains were indistinguishable or very similar to each other by rep-PCR, but different from reference strains *B. petrii* NCTC 13363 and ATCC BAA-461.

PFGE analysis revealed that strains *B. petrii* 1 and *B. petrii* 2 were indistinguishable from each other, while *B. petrii* 3, *B. petrii* 4 and *B. petrii* 5 showed one-band differences indicating that they were related, but not identical (Fig. 1B). Both typing methods suggested higher relatedness of our strains to strain NCTC 13363 than to the type strain ATCC BAA-461.

Following genotypic and phenotypic differences noted among the serial strains, we sought to compare their growth rates. *B. petrii* 1 and *B. petrii* 2 showed identical growth pattern (Fig. 1C, only *B. petrii* 1 shown). In contrast, *B. petrii* 3 grew to a higher CFU/ml, which was evident from 9 hours onwards. The fastest generation time for *B. petrii* 1 and *B. petrii* 2 was ∼1.3 h and that for *B. petrii* 3 was ∼1.45 h (between 3 and 6 h in the growth curve). *B. petrii* 4 displayed slower growth (or a longer lag phase) early (0–9 h) but comparable growth to *B. petrii* 1 and *B. petrii* 2 during later phase of growth (not shown), with generation times of 1.82 h (between 3 and 6 h in the growth curve) and 1.46 h (between 6 and 9 h). Remarkably, *B. petrii* 5 showed consistently diminished growth throughout most of the experiment with generation times of 4.3 h (between 3 and 6 h in the growth curve), 2.6 h (between 6 and 9 h) and 1.29 h (between 9 and 12 h).

### Adaptive Immune Response of the Patient to *B. petrii*

**Figure 2:**
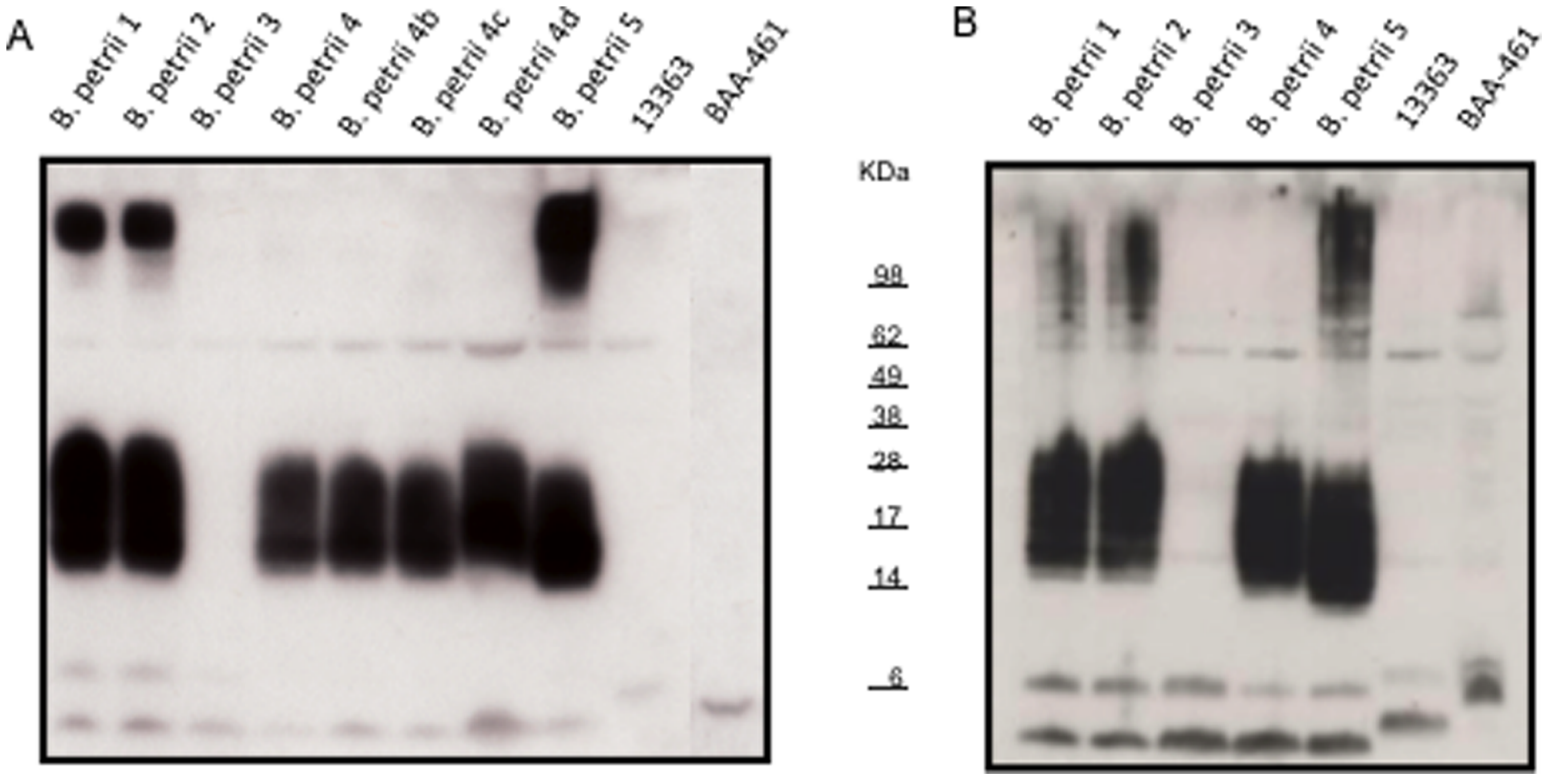
Immunoblots with patient’s serum against her own and reference strains. An aliquot containing 10 µg of protein from the soluble (A) and insoluble (B) fractions obtained from *B. petrii* strains was electrophoresed on 12% SDS–PAGE, and then transferred to PVDF membrane. The membrane was incubated with the patient serum (1∶5000 dilution) obtained ∼2 months after isolation of *B. petrii* 3 and then horseradish peroxidase conjugated sheep anti-human IgG (1∶10000). The blots were developed using the enhanced chemiluminescence kit. *B. petrii* 4, *B. petrii* 4b, *B. petrii* 4c and *B. petrii* 4d refer to four different colonies obtained from the primary isolation plate of *B. petrii* 4. Fig. 2B had a shorter exposure time than Fig. 2A, so bands could be better visualized. If using similar exposure times, the intensity of the bands in Fig. 2B is 48% higher than in Fig. 2A (as determined by densitometric analysis).

To determine whether the patient generated antibodies against components of *B. petrii*, immunoblots were performed using serum from the patient. A strong response was seen against soluble fractions of bacterial extracts from *B. petrii* 1, *B. petrii* 2, *B. petrii* 4 and *B. petrii* 5 but not those from *B. petrii* 3 or the reference strains (Fig. 2A). A dominant band with apparent MW ∼14–28 KDa, a high molecular weight band (>98 KDa) and two smaller bands (∼6 KDa) were seen in *B. petrii* 1, *B. petrii* 2, and *B. petrii* 5 profiles. *B. petrii* 3 profile included the two smaller bands but not the dominant 14–28 KDa nor the >98 KDa bands, while only the later was missing in *B. petrii* 4. Screening additional colonies of *B. petrii* 4 yielded comparable results.

Similar results were obtained with immunoblots performed against insoluble fractions of the bacterial extracts (Fig. 2B) but the reaction was stronger than that with the soluble fractions.

### Antibody Response of Mice Immunized with *B. petrii*

**Figure 3:**
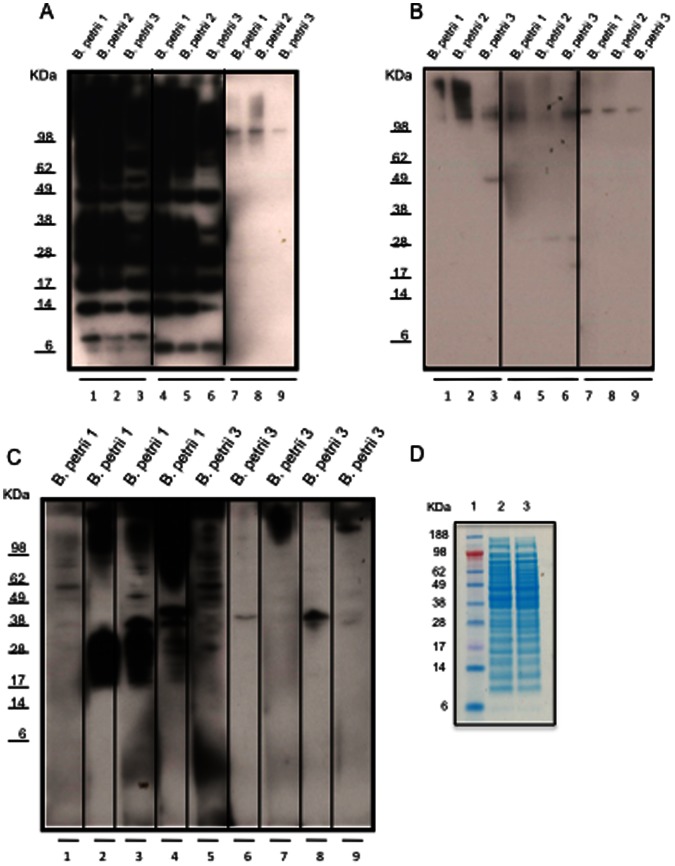
Immunoblots with sera from mice inoculated with *B. petrii* 1 extracts (A), *B. petrii* 3 extracts (B) or live *B. petrii* 1 or *B. petrii* 3 (C) and SDS–PAGE analysis of *B. petrii* 1 and *B. petrii* 3 extracts (D). An aliquot containing 10 µg of bacterial proteins was electrophoresed on 12% SDS–PAGE, and then transferred to PVDF membrane. The membrane was incubated with sera from mice (1∶200 dilution) previously inoculated with extracts from *B. petrii* 1 (3A lanes 1–3 and 4–6 for two different mice) or *B. petrii* 3 (3B lanes 1–3 and 4–6 for two different mice) or with live *B. petrii* 1 (3C lanes 2, 3 and 4 for three different mice) or live *B. petrii* 3 (3C lanes 6, 7, 8 and 9 for four different mice). Uninoculated normal mouse sera controls are shown in 3A lanes 7–9, 3B lanes 7–9 and 3C lanes 1 and 5. The membrane was then incubated with horseradish peroxidase conjugated sheep anti-mouse IgG (1∶10000) and blots developed using the enhanced chemiluminescence kit. (D) *B. petrii* 1 and *B. petrii* 3 protein extracts were analyzed by SDS–PAGE (Invitrogen NuPAGE 12% Bis-Tris gel) and stained with Coomassie Blue R-250 (Invitrogen). Lane 1 Standard (SeeBlue Plus2 pre-stained Standard Invitrogen); lanes 2, *B. petrii* 1 extracts; and lane 3, *B. petrii* 3 extracts (10 µg proteins per lane).

To determine whether the differential antibody recognition of *B. petrii* strains was due to an impaired antibody response and/or changes in the bacteria, extracts from *B. petrii* 1 and *B. petrii* 3 were inoculated ip into wild type mice, and sera collected for immunoblot analysis. Mice inoculated with *B. petrii* 1 (Fig. 3A lanes 1–3 and 4–6) recognized antigens in *B. petrii* 1, *B. petrii* 2, and *B. petrii* 3; however, the response to *B. petrii* 1 and *B. petrii* 2 was stronger. On the other hand, sera from mice inoculated with *B. petrii* 3 (Fig. 3B lanes 1–3 and 4–6), showed less response against all of the strains. Repeat immunoblots using a higher concentration of mouse serum (1∶150) yielded similar results (not shown). Similarly, live *B. petrii* 1 generated a strong antibody response against that strain (Fig. 3C lanes 2–4), while live *B. petrii* 3 showed a poor response (lanes 6–9). *B. petrii* 1 and *B. petrii* 3 protein extracts appeared very similar by SDS-PAGE analysis (Fig. 3D).

### LPS Drives the Adaptive Immune Response in *B. petrii*

**Figure 4:**
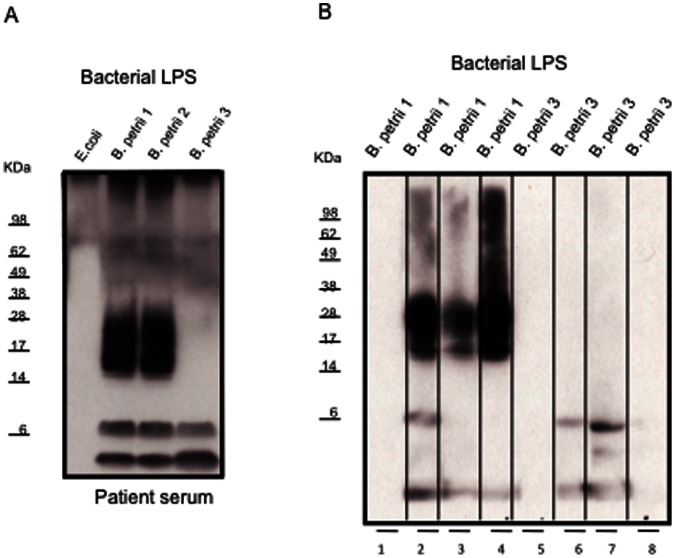
Immunoblots with patient (A) or mice (B) sera against *B. petrii* LPS. *B. petrii* LPS was isolated from bacterial pellets by using a LPS extraction kit. *B. petrii* LPS (10 µg/lane) or commercially obtained *E. coli* LPS control (3 µg/lane) were electrophoresed on 12% SDS–PAGE, and then transferred to PVDF membrane. The membrane was incubated with the patient serum (1∶5000 dilution) or sera from mice (1∶200 dilution) previously inoculated with *B. petrii. B. petrii* 1 LPS (4B lanes 2, 3 and 4 for three mice) or *B. petrii* 3 LPS (4B lanes 6, 7 and 8 for three mice) or with uninoculated normal mouse sera controls (4B lanes 1 and 5). The membrane was then incubated with horseradish peroxidase conjugated sheep anti-human (A) or anti-mice (B) IgG (1∶10000) and blots developed using the enhanced chemiluminescence kit.

The stronger patient antibody reaction against *B. petrii* antigens in the insoluble fraction suggested a membrane-associated component. To test whether it was LPS, immunoblots were performed with LPS purified from *B. petrii* 1, *B. petrii* 2 and *B. petrii* 3. The patient response to *B. petrii* 1 and *B. petrii* 2 LPS was remarkably stronger than to LPS from *B. petrii* 3 (Fig. 4A), recapitulating what was observed with bacterial extracts. Purified LPS from *B. petrii* 1 and *B. petrii* 3 was inoculated into mice and sera collected for immunoblots experiments. As shown in Fig. 4B, *B*. *petrii* 1 LPS elicited a good antibody response in mice, while the response to *B. petrii* 3 LPS was poor (75% higher intensity of *B. petrii* 1 LPS compared to *B. petrii* 3 LPS by densitometric analysis).

### *B. petrii* 3 is a Spontaneous Mutant with a Defective LPS

**Figure 5:**
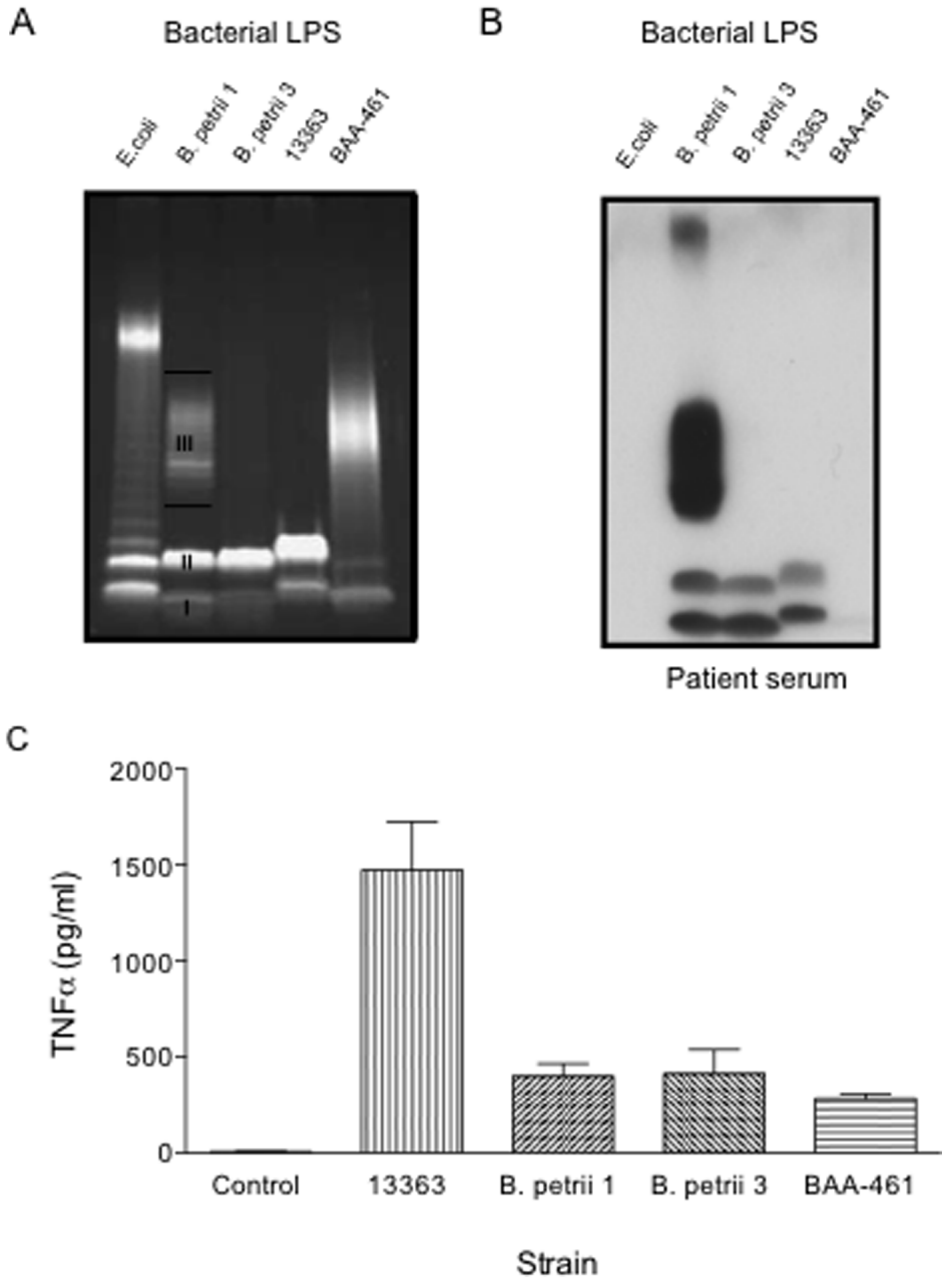
SDS PAGE (A), immunoblots with our patient’s serum (B) and TNF-α induction of *B. petrii* LPS. (A) Five ml overnight cultures from each bacterial strain (*B. petrii* 1, *B. petrii* 3, NCTC 13363 and ATCC BAA-461) were spun 10 min at 8000g to harvest bacterial cells and LPS isolated by using a LPS extraction kit. Extracted LPS samples were separated on a 12% SDS-PAGE and stained using Pro-Q Emerald 300 Lipopolysaccharide gel stain kit. LPS concentrations were measured spectrophotometrically at 205 nm. Control *E coli* LPS was obtained commercially. I, II and III indicate LPS bands I, II and III respectively, described in the text (B) Immunoblots of our patient’s serum against LPS were performed as described in Fig. 4A. (C) Purified LPS (200 ng/ml) was added to human peripheral blood mononuclear cells (PBMCs) from normal volunteers, and supernatants were collected for cytokine measurements after 20 hours. *p*<0.001 for NCTC 13363 vs *B. petrii* 1, *B. petrii* 3 or BAA-461. Graph shows mean and SEM from three experiments.

Following the differential antibody recognition of *B. petrii* 3 LPS by the patient and inoculated mice, we compared the SDS-PAGE profiles of LPS from *B. petrii* 3, *B. petrii* 1 and reference strains. As shown in Fig. 5A, the LPS from *B. petrii* 1 and *B. petrii* 3 show similar profiles for the putative lipid A-inner core (band I) and lipid A-inner core-outer core (band II) components. However, while *B. petrii* 1 LPS shows a band corresponding to the lipid A-inner core-outer core-O-antigen (band III), the *B. petrii* 3 LPS lacks this component. The environmentally isolated type strain of *B. petrii* (ATCC BAA-461) has a band suggestive of O-antigen, while the strain NCTC 13363 does not (Fig. 5A). Interestingly, patient serum recognized putative bands I and II in the LPS of NCTC 13363, but failed to recognize any band of ATCC BAA-461 (Fig. 5B). As expected, the patient recognized O-antigen band of *B. petrii* 1 LPS as well as band I and II. The later were also detected in *B. petrii* 3 LPS.

Endotoxin and proimmflammatory activity were assessed to further characterize LPS samples. *B. petrii* 1 and *B. petrii* 3 LPS showed comparable endotoxin activity with the lowest detectable level at the same LPS concentration (0.8 ng/ml). As seen in Fig. 5C, NCTC 13363 LPS showed the highest TNF-**α** induction. As expected, *B. petrii* 1 and *B. petrii* 3 LPS induced comparable levels of TNF-**α** to one another, which was higher than that of the type strain, although this difference was not significant? *B. petrii* 1 and *B. petrii* 3 LPS also induced comparable levels of other proinflammatory cytokines such as IL-1β and IL6 (data not shown).

**Figure 6:**
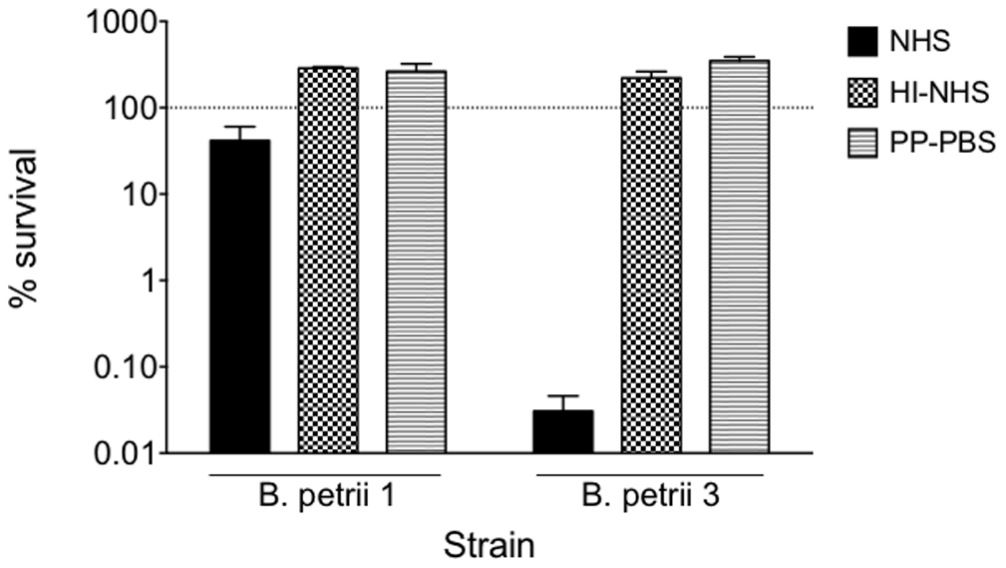
Serum susceptibility of *B. petrii* 1 and *B. petrii* 3. Bacterial colonies grown on SBA were resuspended in 1% proteose peptone - phosphate-buffered saline (PP-PBS) to a concentration of 1×10^7^ CFU/ml. A 100-µl aliquot was then combined with an equal volume of 10% normal human serum (NHS) diluted in 1% PP-PBS. A 1% PP-PBS (0% serum) solution and heat-inactivated (56°C for 1 hour) normal human serum (HI-NHS) served as controls. Samples were incubated for 2 hours at 37°C with shaking. After incubation, samples were serially diluted, plated onto sheep blood agar plates, and grown 24–48 h at 37°C to determine the number of CFU and calculate % of survival.

Since O-antigen plays an important role in resistance to complement-mediated serum killing, we sought to compare the serum sensitivity of O-antigen containing *B. petrii* 1 with O-antigen deficient *B. petrii* 3. As seen in Fig. 6, *B. petrii* 3 was dramatically more susceptible to normal human serum than *B. petrii* 1 with only 0.03% survival compared to 41% for *B. petrii* 1. Serum sensitivity was not observed with heat-inactivated serum indicating that this effect was complement dependent.

### Sequencing Analysis of Patient Strains

Since *B. petrii* 3 was related but distinct from earlier strains and also showed an O-antigen deficient LPS, we sought to find a mutation for this phenotype. Genomic DNA from *B. petrii* 1, *B. petrii* 3, *B. petrii* 4, and *B. petrii* 5 was sequenced (>17x) and draft genome assemblies produced. For three assemblies (*B. petrii* 1, *B. petrii* 3 and *B. petrii* 5), surprisingly low contig N50 values were noted (∼5 kb; 1700–2500 contigs). Assembly was most successful for *B. petrii* 4 (N50 ∼30 kb; 535 contigs).

The *B. bronchiseptica wbm* locus [8] was used to search the draft genomes. Alignment with finished *Bordetella* genomes matched genes flanking the putative LPS biosynthesis operon (e.g., *aspS* and *waaC*), but not O-antigen biosynthesis genes (by BLASTN). Using the closest genome (*B. petrii* ATCC BAA-461) as reference, our sequenced strains were found to be missing the ∼28 kb sequence encoding *Bpet4814*-*Bpet4837*, which include LPS biosynthesis genes. This sequence was replaced with a ∼12 kb sequence predicted to encode ten genes including an O-antigen polymerase, sugar transferases, a transport system and hypothetical proteins (Fig. S1).

Multiple alignments of this putative O-antigen locus from *B. petrii* 1, *B. petrii* 3, *B. petrii* 4, and *B. petrii* 5 identified a single 11 base-pair deletion in *B. petrii* 3 in a gene predicted to encode a ∼260 amino acid glycosyltransferase, family-2 protein. This protein matches (BLASTP) *Mesorhizobium* glycosyltransferase YP_676231.1 (51% identity) followed by similar proteins from *Francisella* and *Clostridium* spp. The deletion and resulting frameshift mutation disrupts the last 10 residues: …KRCAWCGMFQX, which are replaced by 19 different residues (Fig. S1). Very strikingly, Sanger sequencing revealed the same 11-base deletion in the same glycosyltransferase of O-antigen deficient strain NCTC 13363.

## Discussion

We have studied the evolution of an 8-month infection with *B. petrii*. Like previously reported clinical strains, our strains only showed ∼99% similarity to the environmental type strain by 16S rDNA [6], [10], [12]. Our strains (*B. petrii* 1-*B. petrii* 5) were clonally related but showed distinct PFGE profiles underlying genomic differences, which we hypothesize, occurred *in vivo*. Interestingly, *B. petrii* colony variants have been shown to occur *in vitro* linked to genomic rearrangements [20]. Recently, Le Coustumier *et al.* noted PFGE patterns of serial *B. petrii* strains and described these as “quite similar” but no specific phenotypic or genotypic differences were identified [11] suggesting no apparent *in vivo* adaptation in their patient.

*B. petrii* 3, *B. petrii* 4 and *B. petrii* 5 displayed smaller-sized colonies than *B. petrii* 1 and *B. petrii* 2 upon primary isolation, which was not due to extended incubation [20] or serial passages. Our strains also showed increasing antibiotic resistance and *B. petrii* 4 and particularly *B. petrii* 5 displayed reduced growth rates; neither of these phenotypic changes has been reported previously for *B. petrii*. Reduced growth rate may highlight a reduction in bacterial fitness, associated with antibiotic resistance mechanisms [21].

Similar to the *B. petrii* strains described by Le Coustumier *et al* [11], Fry *et al.* [6] and Stark *et al.* [10], our strains were resistant to cephalosporins. Like the first strain (FR 3799) from the patient reported by Le Coustumier *et al* [11], *B. petrii* 1 had low MICs for imipenem, gentamycin, tobramycin, piperacillin/tazobactam and TMP/SMX and appeared resistant to ciprofloxacin; unlike FR 3799, *B. petrii* 1 was susceptible to levofloxacin and meropenem. While the patient described by Le Coustumier *et al*, was treated with amoxicillin/clavulanate, our patient received multiple drugs to treat her MI and *B. petrii* infections. Meropenem and TMP/SMX were specifically added at different times to treat *B. petrii*, and resistance was subsequently observed for both drugs.

Immunoblots against bacterial extracts show a highly specific response of the patient against her strains as compared to NCTC 13363 and the type strain (Fig. 2) or *B. bronchiseptica* (not shown). These immunoblots also showed a differential response to *B. petrii* 3 versus the patient’s other strains. In contrast, using immunoblots against bacterial suspensions, Le Coustumier *et al.* showed that sera from a *B. petrii*-infected patient recognized her own *B. petrii* strains (with identical profiles) but also unrelated clinical and environmental strains as well as other *Bordetellae* [11].

Mice studies highlight the role of antibodies in clearing *Bordetella* infections [22]. Here, antibodies from mice inoculated with *B. petrii* 1 extracts (Fig. 3A) reacted with more components in immunoblots than those inoculated with either live *B. petrii* 1 (Fig. 3C) or *B. petrii* 1 LPS (Fig. 4B), both of which resembled the patient response to natural infection (Fig. 2). Despite a detectable antibody response in our patient, there was no clearance of the infection. Infection persistence despite a detectable antibody response has been described for *B. parapertussis* and *B. pertussis* [1], [22].

*B. petrii* 3 or its extracted components failed to generate a strong immune response in mice (Fig. 3 and 4). *B. petrii* 3 antigens were only recognized by mice previously inoculated with *B. petrii* 1 extracts (Fig. 3A), suggesting that this response was against antigens shared between the two strains. Our study points to O-antigen containing LPS as the major immunogenic component in *B. petrii*, as seen with *B. parapertussis* [23]. We hypothesize that *B. petrii* 3 is an O-antigen deficient mutant of *B. petrii* 1. *B. petrii* 1 and *B. petrii* 3 LPS both showed comparable endotoxin and proinflammatory activities, which are related to lipid A [24]. In contrast, O-antigen deficient *B. petrii* 3 was dramatically more susceptible to human serum than *B. petrii* 1, in agreement with the protective role of *Bordetella’s* O-antigen against complement-mediated killing [25], [26].

Genomic analysis indicates that our strains have swapped the Bordetellae O-antigen biosynthesis cluster for a different system. Intra- and inter-species transfer of O-antigen genes has been described in different bacteria [27], [28], [29], including *B. bronchiseptica* [30]. Importantly, O-antigen loss in *B. petrii* 3 is not due to a complete deletion or disruption of the LPS biosynthesis locus. Instead, we identified a single 11 base-pair deletion in a putative glycosyltransferase. We hypothesize that this mutation disrupts the folding, stability or localization of the protein, perhaps by deleting a pair of cysteine residues (Fig. S1). Due to highly fragmented draft genomes, we cannot exclude the possibility of additional mutations.

This clinical case with serial strains highlights the impressive capacity of *B. petrii* to undergo rapid changes presumably while in the host, possibly due to its genomic plasticity [20]. The O-antigen deficient *B. petrii* 3 exemplifies a specific bacterial population selected during the course of the infection. We hypothesize that it was later eliminated due to its serum and/or antibiotic susceptibility or remained in a protected niche (i.e. intracellularly and/or within a specific organ or anatomic location). It is possible that *B. petrii* infection occurs in locations protected from the antibody response (i.e. tooth abscess) [6], or in immunocompromised patients as shown here.

It is remarkable that two independently isolated distinct clinical strains of *B. petrii* not only had an O-antigen deficient LPS but also the same deletion in the same glycosyltransferase in the putative O-antigen biosynthesis locus. Although this region does not seem to be within the excisable genomic islands of BAA-461 [20], a similar genomic rearrangement may have occurred in both strains leading to O-antigen loss and escape from antibody recognition.

Despite this patient’s complex clinical presentation, an important role for *B. petrii* in her disease course is supported by clinical and radiographic improvements concomitant with the treatment of *B. petrii* despite only minor changes in her mycobacterial cultures. Her condition repeatedly worsened in relation to repeated *B. petrii* isolation. A genetically distinct *B. petrii* isolated from a postmortem spleen specimen supports the presence of different niches in the patient with distinct bacterial populations. Most importantly, it provides evidence of dissemination of the infection beyond the respiratory tract, suggesting a more pathogenic potential for *B. petrii* than previously recognized.

## Supporting Information

Figure S1 A) Mauve alignment of the LPS region from *B. petrii* ATCC BAA-461 (top) to the homologous region of *B. petrii* 4 (bottom). The red trace is sequence similarity. Gene locations are marked as boxes. The region of difference is bounded in a blue box. B) Predicted genes in the putative *B. petrii* 4 LPS operon. The glycosyl transferase that is interrupted in *B. petrii* 3 is marked (*). C) Alignment of the marked (*) glycosyl transferase, family 2 protein C-terminus in *B. petrii* 4 and *B. petrii* 3
(TIF)
