# Supplementary figures and images for: Adaptability and Persistence of the Emerging Pathogen Bordetella petrii
Source: PLoS One. 2013 Jun 4;8(6):e65102. doi: 10.1371/journal.pone.0065102 (PMC3672207; doi:10.1371/journal.pone.0065102)

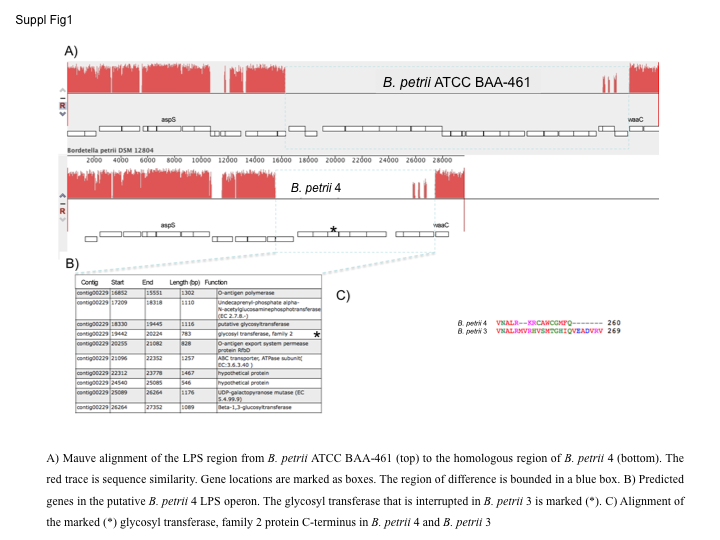

Supplement: Figure S1 — A) Mauve alignment of the LPS region from B. petrii ATCC BAA-461 (top) to the homologous region of B. petrii 4 (bottom). The red trace is sequence similarity. Gene locations are marked as boxes. The region of difference is bounded in a blue box. B) Predicted genes in the putative B. petrii 4 LPS operon. The glycosyl transferase that is interrupted in B. petrii 3 is marked (*). C) Alignment of the marked (*) glycosyl transferase, family 2 protein C-terminus in B. petrii 4 and B. petrii 3 (TIF) [file pone.0065102.s001.tif]
